# Update of the recommendations for the determination of biomarkers in colorectal carcinoma: National Consensus of the Spanish Society of Medical Oncology and the Spanish Society of Pathology

**DOI:** 10.1007/s12094-020-02357-z

**Published:** 2020-05-16

**Authors:** P. García-Alfonso, R. García-Carbonero, J. García-Foncillas, P. Pérez-Segura, R. Salazar, R. Vera, S. Ramón y Cajal, J. Hernández-Losa, S. Landolfi, E. Musulén, M. Cuatrecasas, S. Navarro

**Affiliations:** 1 Departament of Medical Oncology, Hospital General Universitario Gregorio Marañón, Madrid, Spain; 2 Departament of Medical Oncology, Hospital Universitario 12 de Octubre, Instituto de Investigación Sanitaria Hospital 12 de Octubre (imas12), UCM, CNIO, CIBERONC, Madrid, Spain; 3 Departament of Medical Oncology, Hospital Universitario Fundación Jiménez Díaz, Universidad Autónoma de Madrid, Madrid, Spain; 4 Departament of Medical Oncology, Hospital Clínico Universitario San Carlos, CIBERONC, Madrid, Spain; 5 Departament of Medical Oncology, ICO L’Hospitalet, Oncobell Program (IDIBELL), CIBERONC, Hospitalet de Llobregat, Spain; 6 Departament of Medical Oncology, Complejo Hospitalario de Navarra; Navarrabiomed, IDISNA, Pamplona, Spain; 7 Department of Pathology, Hospital Universitario Vall D’Hebron, CIBERONC, Barcelona, Spain; 8 Department of Pathology, Hospital Universitari General de Catalunya, Grupo Quirónsalud, Sant Cugat del Vallès, Spain; 9 Cancer Epigenetics Group, Institut de Recerca Contra La Leucèmia Josep Carreras, Badalona, Spain; 10 Department of Pathology, Hospital Clinic, CIBERehd, Barcelona, Spain; 11 Department of Pathology, University of Valencia, Hospital Clínico Universitario de Valencia, CIBERONC, Valencia, Spain

**Keywords:** Diagnosis, Neoplastic disease, Gastrointestinal, Genetic prognosis, Tumour

## Abstract

In this update of the consensus of the Spanish Society of Medical Oncology (Sociedad Española de Oncología Médica—SEOM) and the Spanish Society of Pathology (Sociedad Española de Anatomía Patológica—SEAP), advances in the analysis of biomarkers in advanced colorectal cancer (CRC) as well as susceptibility markers of hereditary CRC and molecular biomarkers of localized CRC are reviewed. Recently published information on the essential determination of *KRAS*, *NRAS* and *BRAF* mutations and the convenience of determining the amplification of human epidermal growth factor receptor 2 (HER2), the expression of proteins in the DNA repair pathway and the study of *NTRK* fusions are also evaluated. From the pathological point of view, the importance of analysing the tumour budding and poorly differentiated clusters, and its prognostic value in CRC is reviewed, as well as the impact of molecular lymph node analysis on lymph node staging in CRC. The incorporation of pan-genomic technologies, such as next-generation sequencing (NGS) and liquid biopsy in the clinical management of patients with CRC is also outlined. All these aspects are developed in this guide, which, like the previous one, will remain open to any necessary revision in the future.

## Introduction

During recent years, there have been advances related to the analysis of biomarkers with diagnostic, prognostic and therapeutic value in colorectal cancer (CRC), especially in advanced disease. Five years have passed since the last review of the consensus guide of the Spanish Society of Medical Oncology (Sociedad Española de Oncología Médica—SEOM) and the Spanish Society of Pathology (Sociedad Española de Anatomía Patológica—SEAP) [1], and it is important to update it to incorporate the advances that facilitate the administration of precision therapies to these patients.

In addition, this review examines the novelty of hereditary CRC susceptibility markers and the molecular biomarkers of localized CRC. Recently published information on the essential determination of *KRAS*, *NRAS* and *BRAF* mutations is also evaluated [2], as well as the convenience of determining other biomarkers, such as the amplification of the epidermal growth factor receptor (HER2), the expression of proteins in the DNA repair pathway and the study of *NTRK* fusions [2].

From a pathology point of view, new data have been published with a high degree of evidence regarding the diagnostic and prognostic value of some biomarkers that will be detailed in this review. One example is the incorporation into pathology diagnostic protocols the analysis of the tumour budding (TB), defined as the presence of isolated tumour cells or small groups of less than five tumour cells at the invasive tumour front, or the poorly differentiated clusters (PDC), defined as groups of five or more tumour cells at the invasive front of the stroma of the CRC which does not form glandular structures. Both, TB and PDC are prognostic factors in CRC [3, 4].

Another important pathological aspect is the introduction of new molecular assays for the analysis of lymph nodes extracted from surgical specimens of CRC. The pooling-OSNA® (one-step nucleic acid amplification) method, based on the RT-LAMP (reverse transcription loop-mediated isothermal amplification) of cytokeratin 19 (CK19) mRNA for the detection of lymph node metastases, surpassing the current performance of conventional morphological analysis with H&E. Molecular lymph node staging is relevant, especially in the early stages of the disease [5].

Finally, this review evaluates the future incorporation of new pan-genomic technologies, such as next-generation sequencing (NGS) and liquid biopsy for the clinical management of patients with CRC. All these aspects are developed in this guide, which, like the previous one, will remain open to any necessary revision in the future.

## Clinical aspects

### Molecular markers of hereditary CRC susceptibility

Of all CRC cases diagnosed each year, 25% present some characteristic indicating hereditary cancer susceptibility. Of these, 5% correspond to Lynch syndrome, 1% to polyposis and the rest to what could be called ‘family aggregations’.

The identification of these clinical pictures has a high impact on the prevention of CRC, given that the identification of incipient neoplastic lesions leads to cure rates of up to 90% and affects comorbidities, quality of life and health efficiency.

#### Lynch syndrome

The identification of this type of syndrome is based on the Amsterdam I and II criteria [6, 7], which are exclusively clinical. When a family complies, genetic testing should be offered to identify the presence of germline mutations in DNA repair genes (mismatch repair [*MMR*] genes), such as *MLH1*, *MSH2*, *MSH6* and *PMS2*. This genetic study should always be accompanied by adequate genetic counselling.

**Fig. 1:**
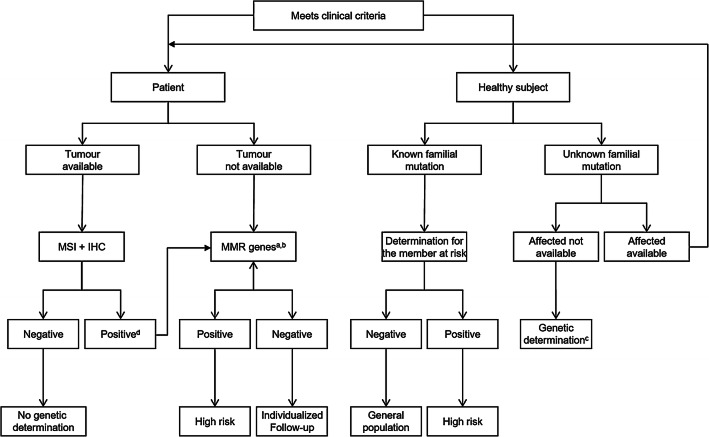
Performance algorithm to detect Lynch syndrome. ^a^Assess at the individual level. ^b^When there is no available tumour, but the Amsterdam criteria are met and the implications for the family are important, the option of conducting a study of germline mutations in *MMR* genes should be evaluated. ^c^When there are no living or available affects, the germinal study should be evaluated in a healthy subject, as long as the impact on family management is important. ^d^In the event that the lack of expression is in *MLH1,* promoter methylation analysis and B6A6 V600E mutation should be performed*.* *IHC* immunohistochemistry, *MMR* mismatch repair genes, *MSI* microsatellite instability

The algorithm for the identification of these mutations is well described and accepted by different scientific societies (Fig. 1). Initial screening is performed by identifying the lack of expression of the corresponding proteins of the genes involved in CRC and/or endometrium or by analysing microsatellite instability [MSI]*.* Any of these techniques is fully accepted today, and each has its specificities. In the case that any of these alterations exist, and once it has been ruled out that it is due to an exclusively somatic alteration, the germinal study of the *MMR* genes should be performed*.*

Notably, at present, this type of study is being performed in any patient with CRC. This measure is justified not only by the preventive implications for the patient and their families but also because it helps to decide whether to administer adjuvant therapy in patients in whom the indication of such treatment is doubtful [8].

#### Polyopsis syndromes

The main characteristic of polyposis syndromes, which usually affect the younger population, is the development of a large number of polyps along the digestive tract, with different histologies and associated with other systemic lesions, with no histological markers to identify them.

If a person and/or their family have a polyposis syndrome, genetic counselling should be offered, and a germinal study of *MMR* genes should be carried out according to the clinical picture.

Taking into account this information, this panel of experts recommends the following:

- Perform MSI and/or immunohistochemistry (IHC) analysis for all patients diagnosed with CRC; and
- Continue with the germinal study of *MMR* genes in patients in which MSI is observed and/or loss of expression of repair proteins, provided that an exclusively somatic origin has been ruled out.

### Molecular markers of localized CRC

The use of adjuvant chemotherapy in patients with stage II CRC is controversial and is only recommended if at least one validated risk factor is observed. Among the clinical, pathological and molecular markers that have been correlated with progression-free survival (PFS) and overall survival (OS), only the evaluation of MSI versus stable microsatellites (MSs), T3 versus T4 and the number of lymph nodes analysed (≥ 12–14 versus < 12–14) have been validated repeatedly in prospective randomized clinical trials [9].

However, in patients with stage III CRC, the prognostic value of MSI is attenuated and is not observed in several large prospective cohort studies. The determination of alterations in *RAS* and *BRAF* has been shown to have prognostic value in recent randomized cohort studies but currently has no clinical utility [10].

Several multigenic tests have been developed with a commercial vision, but their overlap is low, and their clinical application is not guaranteed [11]. These tests have focused on patients with stage II CRC, in whom the evidence for administering adjuvant chemotherapy is conflictive, with the aim of selecting patients with higher risk and, therefore, who may benefit more from chemotherapy. Only two tests, Oncotype DX^®^ and GeneFx^®^ Colon [12], have been validated in cohorts of prospective and randomized patients by multivariate analysis in formalin-fixed paraffin-embedded (FFPE) tumour samples*.* Oncotype DX^®^ is the genetic test with the most evidence and has been developed as a real-time polymerase chain reaction (RT-PCR) multigenic quantitative test*.* Its prognostic value has been observed in four independent prospective clinical trials that randomized patients with stage II CRC, observing hazard ratios (HR) between 1.43 and 1.68 [13–16]. It is important to note that Oncotype DX^®^ maintains its prognostic value when other clinically relevant prognostic factors, such as T4 or MSI, are included in the multivariate analysis. However, its clinical utility is not guaranteed due to the lack of predictive value of the benefit of chemotherapy, as well as the small, but significant, prognostic differentiation between low, intermediate and high risk.

In this context, Immunoscore® has recently emerged as an alternative prognostic biomarker. It has been developed as a quantitative measure of the presence of CD3+ /CD8+ lymphocytes that have invaded a tumour. Both its internal validity and its external clinical validation have been demonstrated in long series of patients with stages II and III CRC through multivariate analysis, including when other factors, such as clinical stage and MSI status, were included in the multivariate analysis.[17]. However, the predictive value of the benefit of chemotherapy is uncertain and lacks independent evidence that demonstrates its prognostic value in stage II CRC, which is only observed when combined with known clinicopathological markers and MSI status.

More promising is the analysis of circulating tumour DNA (ctDNA), through which minimal residual disease can be detected with high prognostic value. If this is validated in the studies of clinical utility that are being carried out, these biomarkers may be the most robust in the near future [18]. Lastly, lack of CDX2 expression confers poor prognosis in stage 2 and may also predict benefit from adjuvant chemotherapy, but this must be confirmed in larger prospective randomized series [19].

### Essential molecular markers of advanced CRC

CRC is a disease characterized by its high heterogeneity, a product of genomic instability and its interaction with multiple exogenous factors that influence carcinogenesis (diet, lifestyle, microbiome, etc.) and that constitutes the exposome. This genomic heterogeneity translates into different models of carcinogenesis and, ultimately, different phenotypes. Some of these genomic alterations can directly influence the selection of treatments; therefore, it is standard practice to determine the mutational state of multiple genes, including the determination of the extended *RAS* mutation, the *BRAF* V600E mutation and the MSI status, which is usually performed by IHC staining for *MLH1*, *MSH2*, *MSH6* and *PMS2*.

#### *RAS* mutations

The main biomarker of clinical utility in metastatic CRC is the mutational state of genes in the *RAS* family (*KRAS*/*NRAS*/*HRAS*). Mutations are present in approximately 50% of cases, the most frequent being those that occur in codons 12 and 13 of exon 2 and that condition a state of cellular expansion.

*RAS* mutations are a predictor of the absence of response to treatment with epidermal growth factor receptor (EGFR) inhibitors, such as cetuximab and panitumumab. In the CRYSTAL study, the addition of cetuximab to the regimen with folinic acid, 5-fluorouracil and irinotecan (FOLFIRI) showed an increase in the response rate (57% versus 40%; *p* < 0.001) and median OS (23.5 versus 20.0 months; HR 0.796; *p* = 0.0093) in patients with tumours without *KRAS* mutations [20]. However, in patients with tumours with *KRAS* mutations, a 4% decrease in the response rate was evidenced, and there were no significant differences in terms of OS [20]. Similar findings were found when analysing the OPUS trial, in which the chemotherapy regimen was folinic acid, 5-fluorouracil and oxaliplatin (FOLFOX) with cetuximab, and in the PRIME trial, in which FOLFOX was combined with panitumumab [21, 22]. Taking into account these data [23], guidelines from the American Society of Clinical Oncology (ASCO), the European Society for Medical Oncology (ESMO) and the National Comprehensive Cancer Network (NCCN) recommend the extended determination of *RAS* mutations before the administration of EGFR inhibitors [8, 24, 25]. The *RAS* analysis should include *HRAS*, *KRAS* and *NRAS*, exons 2 (codons 12 and 13), 3 (codons 59 and 61) and 4 (codons 117 and 146) [8, 24, 25].

#### *BRAF* mutations

The most frequent *BRAF* mutation is the substitution of glutamic acid for valine in codon V600E, which produces constitutive activation of the MAPK pathway. This mutation occurs in 8–10% of patients with metastatic CRC and excludes the *RAS* mutation [26]. It is more common in women, usually appears in more advanced stages and has been associated with tumours of the right colon, poorly differentiated, with mucinous histology and with MSI. Additionally, it seems that *BRAF* mutations lead to the development of more peritoneal metastases and less disease limited to the liver or lung.

Regarding the prognostic role of *BRAF*, it seems that mutation is a negative prognostic factor, although its association with MSI has to be taken into account. A joint analysis of four phase III clinical trials of first-line treatment in patients with metastatic CRC evaluated the possible prognostic value of *BRAF* [27]. In a total of 3063 patients, of whom 8.2% had a *BRAF* mutation, the OS was 11.4 months, and the PFS was 6.2 months, compared to 17.2 months and 7.7 months, respectively, that were observed in patients without *BRAF* mutations (*p* < 0.001). This same study analysed the possible prognostic value of MSI, and it was observed that the OS for patients with MSI of high instability (MSI-H) was 13.6 months, while in patients with MSs, it was 16.8 months (*p* = 0.001). When the *BRAF* and MSI statuses were analysed, there were no differences in terms of PFS or OS in the MSI-H population regardless of *BRAF* status*,* but in the MSs population with mutated *BRAF,* a significant decrease in OS and PFS was observed. These data support the negative prognostic value of *BRAF* mutations.

Recent data support the predictive value of *BRAF* mutations [28]. Phase III BEACON Colorectal Cancer Study enrolled 665 patients with *BRAF* V600E across three cohorts, including the triplet regimen of encorafenib, binimetinib and cetuximab; the doublet regimen of encorafenib and cetuximab; and a control cohort where patients could receive either cetuximab and irinotecan or cetuximab with FOLFIRI. At the time of the interim analysis, the median duration of follow-up was 7.8 months. The median OS was 5.4 months, 8.4 months and 9.0 months for the control group, the doublet regimen arm, and the triplet regimen arm, respectively. Comparing the results obtained in the triplet regimen arm with the control group, HR was 0.52 (*p* < 0.001). An independent central review committee assessed the response rate in this analysis for the first 331 randomized patients. The response rate in the control arm was 2% compared with 20% in the doublet regimen arm and 26% in the triplet regimen arm (control arm versus triplet regimen arm; *p* < 0.001).

#### MSI

Mutations in the *MLH1, MSH2, MSH6* and *PMS2* genes are related to poor *MMR* as well as MSI. They appear in 15% of stage II and III tumours but in only 4% of stage IV tumours. In stages II–III, MSI is related to a better prognosis, and specifically in stage II, the better prognosis occurs in the absence of benefits from adjuvant treatment with fluoropyrimidines. However, MSI in stage IV is associated with an unfavourable prognosis, most likely due to its association with *BRAF* mutations.

Two recent studies show that the alteration of *MMR* may have a predictive role in response to immunotherapy; therefore, the NCCN recommends its determination in all patients with metastatic CRC [29, 30]. The Food and Drug Administration (FDA) has approved the use of nivolumab and pembrolizumab in patients with *MMR-*deficient and MSI-H metastatic CRC.

### Recommended molecular markers of advanced CRC

HER2 is overexpressed in a small proportion of CRC. The Cancer Genome Atlas (TCGA) project identified somatic amplifications or mutations of HER2 in 7% of CRC cases and in 5% of the non-hypermutated CRC subgroup (4% amplification, 3% mutation and 1% both), being more frequent in native *RAS* tumours (8%) [31]. An analysis of 3256 patients with CRC from different clinical trials (QUASAR, FOCUS and PICCOLO) confirmed a higher incidence of HER2-positive CRC in native *KRAS/BRAF* tumours and advanced stages [32]. Some studies indicate that the amplification of HER2 is more frequent in distal tumours (PETACC-3 and HERACLES-A), but others have not confirmed that observation (PETACC-8) [33]. Its association with survival is also controversial [32, 33].

Protein overexpression and gene amplification are easily detectable by IHC or fluorescent in situ hybridization (FISH), respectively. The criteria developed and validated in the HERACLES programme defined HER2-positive tumours as tumours with an IHC staining intensity of 3+ in more than 50% of cells or a staining intensity of 2+ with a HER2:CEP ratio (chromosome enumeration probe) greater than 2 per FISH in more than 50% of the cells [34]. Amplification of HER2 is involved in innate and acquired resistance to anti-EGFR drugs [33]. The dual inhibition of HER2 (lapatinib and trastuzumab or pertuzumab), but not the simple block, is capable of inducing durable responses in patient-derived xenograft (PDX) models of native *RAS*/*BRAF*/*PI3K* cetuximab-resistant CRC [33]. Consistent with this, the HERACLES-A trial documented a response rate of 30% in 27 patients with native *KRAS* CRC, positive HER2, and anti-EGFR resistance treated with lapatinib and trastuzumab [35]. Similarly, in the MyPathway Phase II trial, a response rate of 38% was observed in 37 patients with HER2-positive metastatic CRC treated with trastuzumab and pertuzumab [36]. Other strategies being explored are anti-HER2 monoclonal antibody–drug conjugates, such as trastuzumab-emtansine (HERACLES-B and RESCUE assays), and trastuzumab-deruxtecan, at a 25% response rate documented in a phase I study in 12 patients with HER2-positive CRC [37]. The determination of HER2 may, therefore, be of clinical utility and could be justified at least in native *RAS* tumours resistant to anti-EGFR treatment.

The constitutive activation of different kinases as a consequence of gene translocations plays an essential role in the tumourigenesis of multiple neoplasias, including a subgroup of neoplasias of the colon and rectum, and their pharmacological inhibition constitutes one of the greatest therapeutic successes in the field of precision oncology. In a recent series, in which 18,407 tumour samples and 513 ctDNA samples from CRC patients were characterized by massive sequencing, genetic rearrangements of potentially treatable kinases were identified in 126 tumours (0.68%) and seven ctDNA samples (1.36%) [38]. The most frequently identified fusions in this series involved the *RET* (0.15%), *BRAF* (0.12%), *NTRK1* (0.14%) and *ALK* (0.09%) genes. Ninety percent of the tumours with these gene rearrangements were native *KRAS*, with a high proportion of MSI-H: 86.4%, 45.5% and 14.3% of the tumours with *NTRK1*, *RET* and *ALK* fusions, respectively. The presence of MSI or the absence of *RAS* mutations may, therefore, help to better select the subgroup of patients in which it is more profitable to proceed to the molecular screening of these gene alterations.

These fusions can be identified with different methods. Classically, the detection of *ALK* or *ROS1* has been performed using FISH or RT-PCR, but there is a growing trend in the use of mass DNA sequencing techniques, which has the advantage of allowing the simultaneous evaluation of multiple genes with potential utility as therapeutic targets. Other useful techniques include complete or directed sequencing of tumour RNA, sequencing of circulating DNA or IHC (generally used as screening).

The presence of gene rearrangements in *ALK*, *ROS1* or *NTRK* has been associated with worse survival (15.6 versus 33.7 months for patients with [*n* = 27] or without [*n* = 319] tumour gene fusions, respectively) [39]. *RET* fusions have also been associated with worse survival in a series of 24 positive *RET* patients [40]. In November 2018, the FDA approved the specific TRK inhibitor larotrectinib for the treatment of tumours with NTRK1/2/3 fusions, with an overall response rate of 75% (including two of three patients with CRC) and a median duration of response and PFS not attained after a median follow-up of 9.4 months (PFS at 1 year: 55%) [39]. This is the second tumour-agnostic approval in the history of antineoplastic therapy after approval of pembrolizumab for MSI tumours. Entrectinib was approved in August 2019 for the treatment of tumours with NTRK fusions.

## Pathological aspects

### What is relevant in the pathology diagnosis of CRC?

#### Tumour budding (TB) and poorly differentiated clusters (PDC)

The most important prognostic factors in CRC according to the American Joint Committee on Cancer (AJCC) and the 8th edition of the International Union Against Cancer (UICC) are (i) TNM stage, (ii) venous, lymphatic or perineural invasion and (iii) discontinuous tumour deposits [41]. The determination of TB has been incorporated into the pathology diagnosis protocol of the College of American Pathologists (CRC) v4.0.0.1 and the NCCN guidelines v2.2019. Other factors, such as type, histological grade, the configuration of the tumour border and intratumoural inflammatory infiltrate, influence the prognosis and treatment of patients. Tumour budding or TB, defined by the presence of isolated tumour cells and/or groups of less than five cells in the stroma of an invasive front of the tumour, is the morphological evidence of the epithelial-mesenchymal transition process and is an independent factor of poor prognosis in CRC. TB is relevant in different clinical contexts: (i) in pT1 CRC as an independent predictor of lymph node metastasis, (ii) in stage II CRC predicts recurrence risk and contributes to therapeutic management [42, 43], and (iii) intratumoural TB in endoscopic biopsies allows the individualization of neoadjuvant treatment in rectal carcinomas [44, 45]. The International Tumour Budding Consensus Conference (ITBCC) agreed to count TB with HE at the area of the tumour front with the most density using a 20× objective and a field of 0.785 mm^2^. TB is classified as Bd1-low (0–4 buds), Bd2-intermediate (5–9 buds) and Bd3-high (10 or more buds). Providing the numerical value of buds [e.g., Bd3 (count 17)] is recommended [46]. TB should be evaluated with caution in medullary carcinomas and can be very difficult to count in tumours with glandular fragmentation with marked acute inflammation. TB must be distinguished from PDCs, defined as groups of five or more tumour cells in the invasive tumour front that do not form glandular structures [4]. TB should not be reported in rectal tumours treated with neoadjuvancy, or in mucinous carcinomas with signet ring cells within mucus lakes. If TB cannot be assessed, it is indicated as no non-assessable with an explanatory note [47, 48].

#### Lymph node staging

Lymph node staging (pN) is an important prognostic factor in CRC, a predictor of relapse and survival and determinant of the therapeutic management of patients. The pN stage is obtained from the analysis of lymph nodes stained with HE, which has low sensitivity for detecting lymph node micrometastases in stages I-II CRC. HE staining analyses less than 1% of lymph node tissue [49–53], resulting in 11–24% false negatives. A meta-analysis concluded that the presence of micrometastases in lymph nodes undetected by HE is associated with worse survival [54]. To improve the sensitivity of lymph node analysis, molecular lymph node staging has been performed, which has shown an improvement in pN staging, with respect to HE.

The OSNA technique detects copies of CK19 messenger RNA (mRNA) in lymph nodes, achieving overstaging in 11–50% of HE-determined pN0 patients. Likewise, the total tumour load (TTL) or number of CK19 copies in the lymph nodes is correlated with classical high-risk factors in CRC, such as pT and pN stage, histologic grade, mucinous or signet ring histology, tumour size, male sex, lymphatic invasion and number of lymph nodes assessed [55, 56]. Recently, TTL has been correlated with prognosis. The incorporation of molecular lymph node staging in CRC will allow to select stage II patients for adjuvant therapy and assist patient management.

### What are the requirements of an optimal sample?

For the study of biomarkers in CRC, a sample with enough amount of well-preserved tumour will guarantee the performance of the analysis and quality of the results.

#### Preservation of tumour material

Currently, the primary source for biomarkers studies are FFPE tumours and normal tissues, stored at room temperature.

Although the use of formaldehyde solution is the universal method of tissue fixation, formaldehyde produces covalent bridges in aqueous solutions and interacts with the -NH2 groups of proteins, forming cross-linked methylene bridges; the tertiary and quaternary conformations of these bridges are reversible (epitope retrieval), and they interact with DNA by forming hydroxy methylene bridges between two amino groups. In addition, treatment with formaldehyde can produce aberrant mutations by the formation of apurinic and apyrimidinic sites, DNA degradation and cross-linking of cytosines and has recently been considered carcinogenic. Therefore, alternative fixatives have been developed to improve the preservation of nucleic acids. Among these, PAXgene^®^ Tissue Fix (PreAnalytix GmbH Hombrechtikon, Switzerland) and glyoxal acid-free (GAF) stand out [57, 58]. The prolonged storage of paraffin blocks at room temperature causes the constant degradation of the nucleic acids regardless of the type of fixative used. To slow this degradation process, the blocks should be stored at 4 ºC or frozen [59].

#### Minimum sufficient quantity

It is crucial to select tumour material through macro- or microdissection to avoid contamination with normal tissue. To guarantee results, a tumour cell content 10% higher than normal cell content is considered optimal, thus avoiding the possibility of false results. Currently, with the latest generation of sequencing technologies, a threshold of 5% could be established.

The ideal study sample contains the greatest possible number of tumour cells. Thus, it is preferable to compare a resected piece of CRC with the content of a diagnostic endoscopic biopsy or cytological material [60].

### Which techniques are most appropriate for the determination of each biomarker?

Table 1 summarizes the most important biomarkers in CRC along with the recommended techniques to evaluate them and whether its performance is optional or mandatory.

**Table 1:** Main biomarkers used in CRC

Biomarker	Technique	Indication
CRC—universal		
MSI	IHC and/or MSI analysis by qPCR, bPCR, NGS	Required
Immunoscore or immunodensity	IHC and/or digital score	Optional
CRC—localized		
MSI	IHC and/or PCR and MSI analysis	Required
Immunoscore or immunodensity	IHC and/or digital score	Optional
CRC—advanced		
MSI	IHC and/or PCR and MSI analysis	Required
Extended *RAS*	KRAS, NRAS	Required
*B-RAF*	V600E V600E2/K/D/R or M	Required Optional
*HER2*	IHC / FISH / SISH	Optional
Rearrangements of: NTRK1; NTRK2; NTRK3	IHC and FISH, NGS, RT-PCR, NanoString®	Optional in MSI, *MLH1* hypermethylation and *RAS WT*
Liquid biopsy	qPCR, dPCR, NGS, cfDNA Idylla	Optional for patient monitorization

*cfDNA* cell fee DNA, *FISH* fluorescent in situ hybridization, *IHC* immunohistochemistry, *MSI* microsatellite instability, *NGS* next-generation sequencing, *PCR* polymerase chain reaction; *bPCR* bridging PCR, *dPCR* digital PCR, *qPCR* quantitative PCR, *RT-PCR* real-time PCR, *SISH* silver in situ hybridization, *WT* wild type

#### RAS mutations

Currently available technologies for mutational analysis of the *KRAS* and *NRAS* genes have not been significantly modified compared to the last guide [61, 62], except for the incorporation of the mutational status of the *BRAF* gene [25], as reflected in Table 2.

**Table 2:** Molecular techniques employed in CRC

Techniques available	Sensitivity (% mutated DNA)	Characteristics
**Mutational study of isolated genes** (***KRAS, NRAS*** **and** ***BRAF***)		
Methods of direct sequencing		
Sanger method	25	Detects any mutation Requires a greater amount of mutated DNA Inexpensive
Pyrosequencing	5–10	Commercial trial available Requires pyrosequencer
NGS	1–5	Commercial trial available Requires specific equipment Requires experience in molecular biology
PCR-fragment analysis	1–5	Commercial trial available
		Requires specific equipment Requires experience in molecular biology
Quantitative RT-PCR		
TaqMan ® PCR	10	Only detects specific mutations No commercial trial Requires real-time thermocycler
Scorpions-ARMS	1	Only detects specific mutations Commercial trial available Requires real-time thermocycler
Mutated allele enrichment techniques		
PNA-LNA PCR *clamp*	0,1–1	Only detects specific mutations Requires non-commercial LNA probes Requires experience in molecular biology
COLD-PCR	0,1–1	Requires experience in molecular biology Can be associated with sequencing and pyrosequencing techniques
PCR–RFLP	5	Only detects mutations that generate a restriction site Commercial trial available
DHPLC	1	Detects any mutation Special equipment required Requires experience in HPLC
HRM	1	Detects any mutation Requires specific equipment Requires experience in molecular biology
PCR + hybridization	1–5	Only detects specific mutations Commercial trial available Requires specific equipment (in the case of hybridization in *arrays* or strips if there is a large volume) Requires experience in molecular biology
Direct techniques for determining ***RAS***		
Point of care	1–5	Only detects specific mutations Requires specific equipment Does not require experience in molecular biology
IHQ *BRAF* (VE1)		Only detects the mutation V600E of *BRAF* Requires specific automation equipment Does not require experience in molecular biology
**MSI study**		
IHQ		Detects mutations in the *MLH1, MHS2, MSH3* and *PMS2* genes Requires specific automation equipment Has internal controls for each staining
PCR + MSI analysis	1–5	Detects MSI alterations Commercial trial available Requires sequencing and experience in molecular biology
NGS	1–5	Available commercial trials pending validation Requires specific equipment Requires experience in molecular biology
**HER2 amplification study**		
IHQ		High concordance with gene amplification in those overexpressed Requires specific automation equipment Has interpretation guides
In situ hybridization	1–5	Detects amplifications in a specific way Commercial trials available Requires experience and fluorescence microscope (FISH)
NGS	1–5	Available commercial trials pending validation Requires specific equipment Requires experience in molecular biology
***NTRK*** **rearrangements study**		
IHQ		Overexpression associated with the presence of rearrangements of any of the three genes (*NTRK1, NTRK2* and *NRTK3*) Requires specific automation equipment Confirmation with additional molecular techniques
In situ hybridization	1–5	Detects amplifications in a specific way Commercial trials available Requires experience and fluorescence microscope (FISH) and the use of three probes
NGS	1–5	Available commercial trials pending validation Requires specific equipment Requires experience in molecular biology

*ARMS* mutation system refractory to amplification, *COLD-PCR* coamplification at lower denaturation temperatures, *DHPLC* denaturing high-performance liquid chromatography, *FISH* fluorescent in situ hybridization, *HPLC* high-performance liquid chromatography, *HRM* high-resolution fusion, *IHC* immunohistochemistry, *MSI* microsatellite instability, *NGS* next-generation sequencing, *PCR* polymerase chain reaction, *PNA-LNA* peptide nucleic acid-blocked nucleic acid, *RFLP* restriction fragment length polymorphisms, *RT-PCR* real-time PCR

A large number of laboratories continue to use molecular techniques based on sequencing as confirmation techniques of mutations (Sanger sequencing and pyrosequencing) or as screening techniques based on NGS, where different platforms allow these determinations to be performed simultaneously in both clinical trials and in research studies as well as in the assistance activities of several molecular pathology laboratories in Europe [63]. However, the vast majority of laboratories have focused their activity on the use of specific assays to detect specific alterations in these genes (*KRAS*, *NRAS* and *BRAF*) using commercial kits by Conformité Européenne-In Vitro Diagnostic (CE-IVD) at the expense of the tests developed by each laboratory (laboratory developed test [LDTs]), whose use is limited in Spain in comparison with other European countries, such as Holland and Germany [64].

Notably, many of the kits used to determine *KRAS*/*NRAS* mutations do not determine *BRAF* mutations simultaneously; therefore, many laboratories have had to modify their workflows as well as combine molecular techniques to analyse these mutations sequentially (e.g., first, *KRAS* gene mutations are analysed, then *NRAS* gene mutations and finally *BRAF* gene mutations), which increases response times, sample manipulation and complexity for obtaining these results.

In the case of the *BRAF* gene, the majority of the mutations focus on the valine amino acid at position 600, which can be affected in multiple ways (V600E/E2/K/D/R or M); however, given that the vast majority correspond to the p. V600E change, this mutation can be detected by a specific antibody (Clone VE1) via IHC. However, although this technique has shown high sensitivity and analytical specificity in different studies [65, 66], confirmation is currently required by a molecular technique.

In recent years, there has been an increase in the use of commercial point of care (POC) techniques that minimize and automate the process of determining gene mutations, significantly reducing response times, reducing the risk of obtaining false positives/negatives and avoiding DNA extraction because analyses can be performed directly with an FFPE sample, with minimum manipulation by the technical staff and with validated results against conventional techniques and NGS [67], even using DNA extracted from paraffin-embedded samples [68].

In the coming years, it is believed that the use of specific algorithms based on the use of artificial intelligence will increase and be used to analyse both radiological and HE images, which will produce *RAS* analyses with greater precision [69, 70].

#### MSI

Other indispensable markers in patients with CRC are *MMR* deficiency and the presence of MSI for identifying patients with Lynch syndrome with prognostic purpose and to predict the response to different immunotherapeutic treatments.

For this purpose, in the majority of pathology laboratories, IHC analysis of the four repair genes (*MLH1, MSH2, MSH6* and *PMS2*) is performed; however, because the encoded proteins act in tandem, different groups have proposed that only IHQ determination of *MLH1* and *MSH2* can be used as a screening technique [71].

In addition, PCR techniques have been used for the detection of MSI. When developing the methodology, we began using a panel of microsatellites (Bethesda panel) composed of markers that detect single base alterations (BAT25 and BAT26) as well as three dinucleotide markers (D2S123, D5S346 and D17S250), but recently, new mononucleotide satellites have been incorporated, which have shown better prediction capabilities than those of previous dinucleotides [72].

Finally, both recently implemented NGS techniques and new POC tools allow the determination of MSI with new satellites included in their panels and are being validated in different studies [73].

#### Alterations in HER2

The determination of the amplification of HER2 in CRC has generated great interest because of its prognostic and predictive value of response to specific treatments because approximately 3% of patients have such alterations [74].

The methods of analysis are the same as those used in breast and gastric cancers, where IHC (HercepTest^®^ or Clone 4B5) stands out, showing great concordance with the gene amplification detected by FISH or in silver in situ hybridization (SISH) [34]. Currently, both the determination of the copy number alterations (CNAs) in a tumour by NGS techniques and the quantification of the levels of HER2 mRNA are being validated in different studies before their implementation in routine healthcare [39].

#### NTRK rearrangements

The rearrangements of the *NTRK1, NTRK2* and *NTRK3 genes* are of special relevance in CRC because, although their prevalence is very low (1.5%) [39], these alterations are associated with the presence of MSI, hypermethylated *MLH1* and native *RAS* [75]. In addition, it is important to mention that they have a great predictive value of response to TRK inhibitors. The classical techniques for the detection of *NTRK* alterations are generally performed by IHC with pan-TRK clones as screening, with subsequent confirmation by different tests (FISH, NGS, RT-PCR or NanoString®); however, each one has its limitations [76].

## What is the current and future role of NGS in CRC?

The implementation of NGS in the study of CRC will allow, in the future, the diagnosis of Lynch syndrome through the mutational study of the *MMR* and *EPCAM* genes [77] and will guide treatment by detecting the mutations of the main genes involved in the pathways of CRC carcinogenesis, such as *RAS* and *BRAF*, as well as the genes associated with targeted treatment, such as *HER2*. In addition, it will be possible to classify CRC into molecular subtypes (CMS1, CMS2, CMS3 and CMS4) with different biological behaviours and therapeutic responses, which will open the possibility of designing personalized treatment [9]. Likewise, NGS will allow the identification of the tumour hypermutation status, which will guarantee the efficacy of immunotherapy [78].

Furthermore, through the study of ctDNA, it will be possible to monitor response to treatment, anticipate the appearance of local recurrence and metastasis, and detect resistance to ongoing treatment [79].

Finally, the possibility of implementing the combination of different molecular tools in the diagnostic routine will also improve the quality of tumour samples [68], without forgetting that IHC continues to be an indispensable diagnostic technique [80].

## What are the current and future roles of liquid biopsy in CRC?

A liquid biopsy is based on the analysis of a biological liquid, usually blood, but also cerebrospinal fluid or urine, to reveal characteristics of a cancer, such as circulating tumour cells (CTCs), free nucleic acids (DNA or RNA), exosomes or tumour-derived platelets. The quantification of CTCs in peripheral blood has been shown to have prognostic value with a threshold level above or below three cells. The possibility of extracting DNA from these cells for NGS has been recently evaluated [81].

The most developed option in liquid biopsy is the study of ctDNA in peripheral blood. The biomarker with the greatest impact on CRC is the mutation in the *RAS* oncogene, including *KRAS* and *NRAS*, in exons 2, 3 and 4, which indicates patients who may be candidates for treatment with anti-EGFR antibodies in case of the native or non-mutated sequence. This technique has been studied using tumour tissue as a comparison. Different publications have confirmed that using high sensitivity molecular techniques [82], the concordance between the analyses performed on tumour tissue and peripheral blood exceeds 90% [83]. Correctly selecting the analysis technique is critical. Liquid biopsy has two very significant advantages compared to tissue biopsy: (i) it indicates the heterogeneity of the tumour and (ii) it is a real-time reflection of the molecular profile, which allows determining the dynamic evolution of the tumour.

Several studies have observed the convenience of using liquid biopsy in the early determination of response in patients with *RAS* mutations, in whom during the course of treatment the basal mutation was not detected, and in patients with a RAS native sequence, in whom during treatment a *RAS* mutation is detected, predicting, presumably, the onset of radiological progression of the disease. The determination of *BRAF* mutations can be another important application of liquid biopsy because it allows monitoring patients with CRC with this alteration [84].

Recent research has opened the way to new uses of liquid biopsy. Some studies have proposed its possible use for the early diagnosis of CRC; however, this option is still very preliminary and, for the moment, lacks an adequate cost-effectiveness relationship as a population screening technique [85]. Finally, the identification of low-incidence molecular alterations in CRC, such as the presence of HER2 amplification or fusions that affect *NTRK,* could be evaluated via ctDNA by mass sequencing platforms [86].

## Conclusions

In recent decades, significant progress has been made in understanding the molecular characteristics of CRC and in the identification of specific mutations that have allowed the development of new prognostic and predictive biomarkers in the different stages of the disease. These advances, together with those achieved through pathological knowledge, have justified a new edition of this guide.

With respect to the susceptibility markers of hereditary CRC, this panel recommends the analysis of MSI and/or IHC in all patients diagnosed with CRC. In addition, the germinal study of *MMR* genes should continue in patients with MSI and/or loss of repair protein expression. In localized stages, the determination of MSI as a prognostic marker is required, especially in stage II CRC. The standardized implementation of genomic platforms is not recommended due to the lack of predictive value of response to chemotherapy.

In patients with advanced CRC, it is essential to determine extended *RAS* mutations, the *BRAF* V600E mutation and the MSI status, which is usually performed via IHC for *MLH1, MSH2, MSH6* and *PMS2*. The determination of extended *RAS* mutations is mandatory before making therapeutic decisions (I-A). The *RAS* analysis should include *HRAS, KRAS* and *NRAS,* exons 2 (codons 12 and 13), 3 (codons 59 and 61) and 4, as negative predictive factors of the anti-EGFR response. The *BRAF* V600E mutation is required due to its recognized negative prognostic value (I-A) and, recently, as a predictive marker of specific biological treatments (cetuximab, encorafenib and binimetinib). The determination of MSI acquires predictive value in advanced CRC for immunotherapy treatment with pembrolizumab or nivolumab (II-B).

Another recommended marker in advanced CRC is the amplification of HER2 as a negative predictive marker of anti-EGFR response and as a response marker for dual anti-HER2 therapy. Its determination is recommended, at least in native *RAS* tumours resistant to anti-EGFR. The *ALK, ROS1* and *NTRK *fusions*,* despite their low incidence, have clinical interest due to recent approval for specific treatments.

The greatest contribution to the genetic study of CRC will come from liquid biopsies, with which minimal residual disease can be detected after surgery. This technique reflects tumour heterogeneity and molecular profiles in real time, reporting the dynamic evolution of the tumour. The implementation of NGS will improve genotypic knowledge, the tumour hypermutation status and molecular subtypes. The combination of different molecular tools in the diagnostic routine will improve the diagnosis using and quality of tumour samples.
